# Effect of a structured nurse-led empowerment protocol on knowledge, practical skills, and self-care behaviours among patients with permanent pacemakers: a quasi-experimental study

**DOI:** 10.1186/s12912-026-05368-3

**Published:** 2026-10-03

**Authors:** Walaa Nady Sayed Kamel, Aml Ramadan Abdelgalil Assaf, Ahmed Mostafa Shehata, Nahla Mohamed Mahmoud Afify

**Affiliations:** 1 Faculty of Nursing, Beni-Suef University, Beni-Suef, Egypt; 2 Faculty of Nursing, Tanta University, Tanta, Egypt; 3 Faculty of Nursing, Kafrelsheikh University, Kafrelsheikh, Egypt

**Keywords:** Empowerment protocol, Permanent pacemaker, Self-care behaviours, Nurse-led intervention, Self-efficacy, Quasi-experimental study

## Abstract

**Background:**

Patients with permanent pacemakers frequently demonstrate insufficient knowledge and inadequate self-care behaviours, which increase the risk of avoidable post-implantation complications. Structured, theory-informed empowerment protocols may improve these outcomes, yet robust evidence from low- and middle-income countries remains limited. Grounded in Bandura’s self-efficacy theory and a patient-empowerment model, this study evaluated the effect of a nurse-led empowerment protocol on knowledge, practical skills, and self-care behaviours among patients with permanent pacemakers.

**Methods:**

A non-equivalent control-group, pretest–posttest quasi-experimental design was used and reported in accordance with the Transparent Reporting of Evaluations with Nonrandomized Designs (TREND) statement. Seventy adults with newly implanted permanent pacemakers were recruited from Tanta University Hospitals, Egypt, between December 2025 and April 2026. Because the two arms were recruited in consecutive calendar periods to prevent educational contamination, allocation was non-random and group membership is confounded with time period. The control group (*n* = 35) received routine hospital care; the study group (*n* = 35) additionally received a structured empowerment protocol (four individual sessions of 30–45 min each, using illustrated Arabic booklets, demonstration and return demonstration, followed by weekly telephone reinforcement during the first month). Three co-primary outcomes were assessed at baseline, immediately post-intervention, and at one-month follow-up: knowledge (Pacemaker-Related Knowledge Questionnaire, 0–20), practice competence (Observational Checklist, 0–50), and self-care behaviour (Self-Care Behaviours Scale, 0–20). Data were analysed using repeated-measures ANOVA (group × time) with Greenhouse–Geisser correction and independent and paired t-tests; a family-wise threshold of α = 0.017 (0.05/3) was applied across the three co-primary group × time tests. Between-group effect sizes and 95% confidence intervals (CIs) were computed.

**Results:**

The study group improved significantly more than the control group on all three co-primary outcomes. Mean knowledge scores rose from 6.51 ± 2.45 to 15.31 ± 3.46 post-intervention (control: 5.57 ± 2.47 to 7.46 ± 5.60; group × time F(1.43, 97.38) = 148.09, *p* < 0.001, η²*p* = 0.685; between-group post-intervention difference 7.85 points, 95% CI 5.67–10.03, Cohen’s d = 1.69). Practice competence improved from 16.29 ± 2.87 to 41.17 ± 10.00 in the study group (control: 14.91 ± 3.33 to 18.54 ± 12.21; F(1.59, 108.12) = 120.34, *p* < 0.001, η²*p* = 0.639; difference 22.63, 95% CI 17.40–27.86, d = 2.03). Self-care behaviour scores increased from 13.63 ± 3.11 to 19.00 ± 3.23 in the study group (control: 12.86 ± 2.30 to 15.00 ± 3.10; F(2, 136) = 49.85, *p* < 0.001, η²*p* = 0.423; difference 4.00, 95% CI 2.52–5.48, d = 1.26). All three interactions remained significant at the family-wise threshold, and the groups did not differ significantly on any recorded baseline characteristic. Gains were partially sustained at one-month follow-up.

**Conclusions:**

In this non-randomised study, participants who received a structured, nurse-led empowerment protocol showed substantial and partially sustained gains in knowledge, practical skills, and self-care behaviours, whereas the corresponding gains observed under routine hospital care alone were minimal. Because the two groups were recruited in consecutive calendar periods rather than randomised concurrently, and because outcome assessment was unblinded, these results are best interpreted as an association observed under a quasi-experimental design rather than as causal evidence equivalent to that of a randomised controlled trial. They nonetheless provide a reasonable basis for evaluating systematic, interactive empowerment programmes—incorporating individualised education and scheduled reinforcement—as an addition to routine post-implantation nursing care, and for testing them in randomised, attention-controlled trials with blinded assessment and longer follow-up.

**Trial registration:**

ClinicalTrials.gov, NCT07743346. Registered on 26 July 2026. Retrospectively registered. Available at: https://clinicaltrials.gov/study/NCT07743346.

## Background

Pacemakers are electronic devices that regulate or stimulate cardiac rhythm to maintain adequate cardiac output. A permanent pacemaker (PM) is used to manage bradyarrhythmias, complete heart block, and other conduction abnormalities [1]. The PM system comprises a pulse generator (battery and circuitry) and one or more leads inserted into the cardiac chambers [2]. Implantation is typically performed under local anaesthesia in a catheterisation laboratory, with leads guided fluoroscopically into the right-sided chambers [1].

After successful implantation, long-term outcomes depend not only on the device but equally on the patient’s adoption of consistent, informed self-care behaviours. Self-care plays a vital role in preventing post-implantation complications including infection, lead dislodgement, and electromagnetic interference and supports optimal device function and quality of life [3]. Nevertheless, many patients demonstrate insufficient knowledge of pacemaker management, leading to poor adherence and avoidable adverse outcomes [4].

Effective patient education for this population should encompass pulse self-monitoring, incision care, activity restrictions, recognition of warning signs, and precautions against electromagnetic interference [5]. Yet in many settings such education is delivered, if at all, as brief verbal discharge instructions unstructured, unreinforced, and rarely accompanied by skills demonstration or verification of comprehension. Prior nurse-led educational and empowerment interventions have improved knowledge and self-efficacy in cardiac [5] and other chronic-disease [6] populations, but they have often been single-domain (knowledge only), have seldom verified psychomotor competence through observation, and have rarely been evaluated with a comparison group in low- and middle-income settings. This leaves a specific and consequential gap: whether a *structured*,* multi-domain*,* skills-verified* empowerment protocol delivered by a single nurse can produce measurable and durable gains in knowledge, observed practice, and self-care behaviour where routine care currently does not.

Approximately three million people worldwide live with a pacemaker, with an estimated 600,000 new implantations annually [7]. In Egypt, contemporary national registry data are scarce, and historical implantation rates have been markedly lower than the rates exceeding 800 per million reported in parts of Western Europe [8], reflecting a substantial disparity in access to device therapy and underscoring the importance of optimising post-implantation care for those who do receive an implant. Patients commonly struggle to adapt to the required lifestyle modifications, underscoring the need for targeted nursing interventions. Rigorous evaluations of structured, nurse-led empowerment protocols in low- and middle-income settings remain scarce, representing the gap this study addresses.

### Theoretical and conceptual framework

This study was grounded in Bandura’s Self-Efficacy Theory [9], complemented by a patient-empowerment model [10] and, for the self-care outcome, Orem’s Self-Care Deficit Nursing Theory [11]. These frameworks were selected because the intervention’s mechanism of action maps directly onto their core constructs.

Bandura proposes that behaviour change is driven by self-efficacy an individual’s belief in their capacity to perform a given behaviour, which is strengthened through four sources: mastery experiences, vicarious learning, verbal persuasion, and management of physiological states [9]. The empowerment protocol was engineered to activate each source: return demonstration of pulse checking and incision inspection provides mastery experiences; the nurse’s demonstration provides vicarious learning; the interactive question-and-answer segment and illustrated booklet provide verbal persuasion and reinforcement; and the private, supportive delivery format helps regulate the anxiety that commonly accompanies a new implant.

The patient-empowerment model frames the nurse’s role as enabling patients to make informed decisions and take active responsibility for their own management, rather than positioning them as passive recipients of instruction [10]. Orem’s theory conceptualises self-care as a learned, deliberate activity and identifies a *self-care deficit* when a person’s demand for self-care exceeds their capacity precisely the state of a newly implanted patient which nursing systems (here, an educative-supportive system) are designed to remedy [11]. Together, these frameworks predict that a structured intervention targeting knowledge, skills, and self-efficacy will translate into improved self-care behaviour, and they provide the mechanism through which the study’s hypotheses are advanced.

### Operational definition

#### Empowerment protocol

A systematic, structured educational programme designed to strengthen patients’ knowledge, practical skills, and self-care behaviours related to permanent pacemaker management [12]. Consistent with the framework above, it addresses three domains.

- **Knowledge**: pacemaker fundamentals, incision care, activity restrictions, electromagnetic-interference precautions, symptom monitoring, and follow-up requirements.
- **Skills**: demonstration and return demonstration of pulse checking and incision inspection (mastery experiences).
- **Self-care behaviour**: fostering self-efficacy and active patient engagement in ongoing management.

### Aim and hypotheses

This study aimed to evaluate the effect of a nurse-led empowerment protocol on knowledge, practical skills, and self-care behaviours among patients with permanent pacemakers. All three outcomes were designated a priori as co-primary; no outcome was ranked above another. We tested the following hypotheses:

- **H1**: Patients in the study group will achieve higher knowledge scores post-intervention than those in the control group.
- **H2**: Patients in the study group will show greater improvement in *observed* practice competence after the intervention than those in the control group.
- **H3**: Patients in the study group will exhibit greater improvement in *self-reported* self-care behaviours post-intervention than those in the control group.

## Methods

### Study design

A non-equivalent control-group, pretest–posttest quasi-experimental design was used. The study is reported in accordance with the Transparent Reporting of Evaluations with Nonrandomized Designs (TREND) statement. Baseline (pre-test) data were collected uniformly for all participants prior to any intervention. Participants were allocated to groups by consecutive calendar period rather than at random: the control group who received standard hospital routine care was recruited and assessed during the first phase (December 2025 to February 2026), after which the nurse-led empowerment protocol was implemented and the intervention group was recruited and assessed during the second phase (February 2026 to April 2026).

Individual randomisation was not feasible in this setting for three reasons. First, the cardiac intensive care unit and cardiac wards at the study hospital operate as open, shared clinical areas in which patients awaiting or recovering from implantation occupy adjacent beds, share visiting areas and routinely discuss their care; concurrent individual randomisation would therefore have exposed control participants to the intervention content through patient-to-patient transmission and through the illustrated Arabic booklet, which patients could not be prevented from sharing. Second, the protocol was delivered by a single nurse, the second author (ARAA), a medical-surgical nursing researcher, working within the same unit, so contemporaneous delivery to a randomly interleaved subset of patients could not have been concealed from the remaining patients or from ward staff. Third, cluster randomisation was not possible because only one implanting centre was available. A sequential (cohort-controlled) design was therefore adopted as the pragmatic alternative. We acknowledge that this choice purchases protection against contamination at the cost of confounding between group allocation and calendar time, and we treat that confounding as the principal limitation of the study.

Because allocation was determined by calendar period, we examined whether the clinical environment changed between the two recruitment phases. After data collection was complete we retrospectively reviewed institutional records covering the whole study period: monthly unit rosters for nursing and medical staffing establishment; the departmental policy manual and the dated patient information leaflet for discharge education; the catheterisation laboratory log for implantation technique, device type and peri-procedural protocols; and departmental meeting minutes for policy, accreditation and quality-improvement activity. No substantive change was identified in any of these domains between December 2025–February 2026 and February–April 2026, and the review was verified by the nursing service director and the head of the cardiology department. Patient case-mix was assessed empirically rather than administratively: the groups did not differ significantly on any baseline sociodemographic (Table 1) or clinical (Table 2) characteristic. This review was retrospective rather than a formal contemporaneous audit and was confined to documented features of the service, so it cannot capture unrecorded variation in the delivery of routine care; unmeasured temporal factors therefore cannot be excluded as contributors to the observed between-group differences.

### Setting

The study was conducted in the Cardiac Intensive Care Unit, cardiac wards, and cardiac outpatient clinics of Tanta University Hospitals, Egypt, between December 2025 and April 2026.

### Participants

**Fig. 1:**
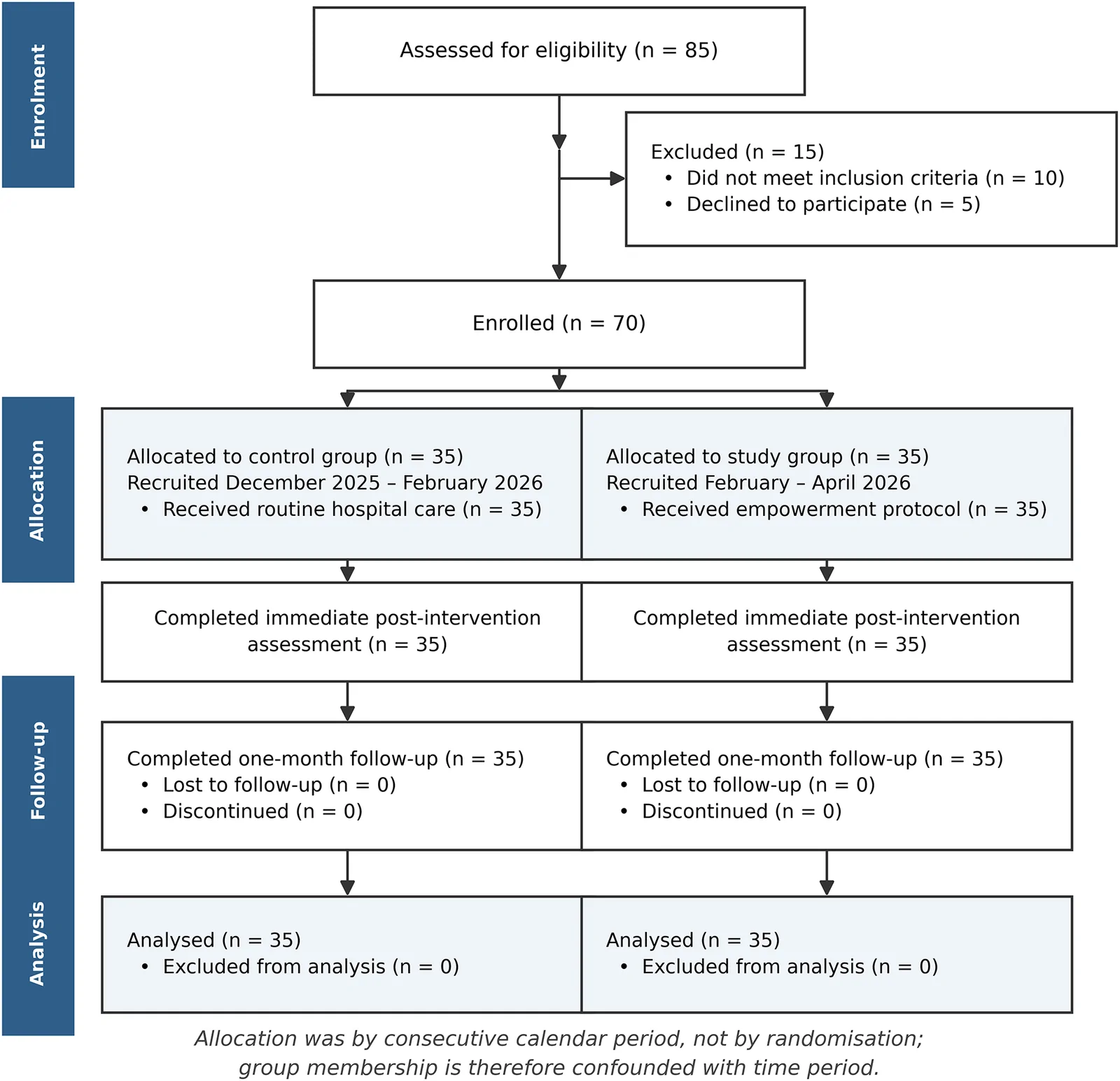
Participant flow through the study, reported in accordance with the TREND statement. Eighty-five adults were assessed for eligibility, 15 were excluded (10 did not meet the inclusion criteria; 5 declined), and 70 were enrolled: 35 allocated to the control group and 35 to the intervention group. All 70 received their allocated condition, completed the immediate post-intervention assessment and the one-month follow-up, and were included in the analysis; none was excluded from analysis

Of 85 consecutive adults screened for eligibility, 70 were enrolled: 15 were excluded, 10 because they did not meet the inclusion criteria and 5 because they declined to participate. Participant flow is reported in full, in accordance with the TREND statement, in Fig. 1. A consecutive (purposive) sampling frame was used within each recruitment period. The sample size was calculated using G*Power 3.1 (test family: F tests; statistical test: ANOVA, repeated measures, within–between interaction; effect size f = 0.40; α = 0.05; power = 0.80; two groups; three measurements; assumed correlation among repeated measures *r* = 0.50; nonsphericity correction ε = 1), yielding a minimum of 30 participants per group; 35 per group were recruited to allow for potential attrition (none occurred). The effect size f = 0.40 was derived from pilot testing with seven participants (three intervention, four control), in which the preliminary between-group improvement in knowledge scores yielded a partial η² of 0.152, corresponding to f = 0.42; f = 0.40 was adopted conservatively. Because the pilot sample was very small, the resulting effect-size estimate is imprecise, and the power calculation should be regarded as indicative rather than definitive. Pilot participants were not included in the main analysis.

Inclusion criteria were: age ≥ 18 years; first-time permanent pacemaker implantation; admission to the cardiology department; and willingness to provide written informed consent.

Exclusion criteria were: documented psychiatric or cognitive disorders; and inability to cooperate with the protocol.

Participants were allocated sequentially, by recruitment period, to minimise educational cross-contamination: the control group was recruited and assessed first, followed by the study group. Group allocation is therefore completely determined by calendar period. This is a recognised source of confounding by history, maturation and secular trend, and is discussed as the principal limitation of the study.

### Intervention

#### Control group

Participants received routine hospital nursing care, comprising verbal discharge instructions and a standard patient information leaflet. They received no attention-matched study contact beyond outcome assessment.

#### Study group

In addition to routine care, participants received the empowerment protocol, delivered individually by the second author (ARAA, a medical-surgical nursing researcher) in a private area, in four sessions of 30–45 min each spanning the peri-implantation period (pre-procedure, post-procedure day 1, post-procedure day 2, and discharge day), as detailed under Data-collection procedure. Each session comprised: verbal instruction supported by visual aids; demonstration and return demonstration of pulse measurement and incision inspection; an illustrated Arabic-language educational booklet; and an interactive question-and-answer segment. Following discharge, intervention fidelity and adherence were reinforced by weekly structured telephone contacts during the first month. Content and sequencing were designed to activate the four sources of self-efficacy described in the framework. Protocol content was validated by five experts (content validity index > 0.85). The control group received none of these contacts; the resulting imbalance in professional attention is addressed in the Limitations.

### Data-collection instruments

Intervention delivery and outcome assessment. The empowerment protocol was designed and delivered by the second author (ARAA), a medical-surgical nursing researcher, who was the sole interventionist throughout the study and delivered every session and telephone contact to the study group. Practical competence (Tool III) was also assessed by the second author (ARAA). The interventionist and the practice assessor were therefore the same individual, and that individual was necessarily aware of group allocation at every assessment. We state this explicitly because it constitutes a major source of measurement bias, and it is identified as such in the Limitations. Outcome assessment was not blinded to group allocation at any time point; because the two groups were recruited in consecutive calendar periods and the protocol was delivered within the same clinical unit, blinding of the assessor was not achievable. A standardised assessment protocol was used throughout: a 50-item observational checklist with explicit, pre-specified behavioural criteria and a binary performed / not-performed rubric for every item, with scoring rules fixed in writing before data collection and applied identically to both groups at all three time points. Because a single assessor performed all practice observations, no inter-rater calibration exercise was possible and no agreement statistic could be calculated. Standardisation was instead achieved by fixing the operational definition and the binary scoring criterion for each of the 50 items in writing before recruitment began, following review by the five content experts who validated the checklist, and by applying these criteria unchanged to both groups at all three time points.

We acknowledge that, because the intervention itself consisted partly of return demonstration of the very skills scored on the checklist, and because the practice outcome was assessed by unblinded direct observation, the potential for observer bias in this outcome is substantial. The explicit, objective scoring rubric reduces but cannot eliminate that risk, and no independent second assessor scored a sample of observations, so inter-rater reliability is unknown. This is identified as a major source of measurement bias in the Limitations.

The observational checklist was completed by direct observation of the patient performing each task: at the bedside on the cardiac unit for the baseline and immediate post-intervention assessments, and at the cardiac outpatient clinic or in the patient’s home for those who did not attend at the one-month follow-up. Each observation took approximately 20–25 min and was scored contemporaneously on the paper checklist rather than from recall. Tools I, II and IV were administered by the fourth author (NMA), who did not deliver any component of the intervention.

#### Tool I – Structured Interview Questionnaire (two parts)

Part A captured sociodemographic data (age, sex, education, marital status, residence); Part B captured clinical data (diagnosis, comorbidities, surgical history).

#### Tool II – Pacemaker-Related Knowledge Questionnaire

20 items across three domains (electrophysiology, pacemaker basics, and care/management). Each correct answer scored 1 point and each incorrect or ‘do not know’ answer 0 (total 0–20). Total scores were categorised as poor (< 12, i.e. < 60%), fair (12–13, i.e. 60–69%) and good (≥ 14, i.e. ≥ 70%), applying the same 70% criterion used for the other two tools. Higher scores denote greater pacemaker-related knowledge.

#### Tool III – Observational Checklist

50 items assessing *observed* self-care practices (wound care, pulse measurement, safety, electromagnetic precautions, medication, and physical activity). Each item was scored performed = 1 or not performed = 0 (total 0–50); higher scores denote greater competence. Competence was defined as ≥ 70% (≥ 35 points). This cut-off was established a priori, before any data were collected, by expert consensus rather than by adoption from a published source: it was not taken from previous literature and it does not correspond to a published minimal clinically important difference, because no such value has been established for this instrument. The five content experts in critical care and medical-surgical nursing who validated the checklist judged 70% to be the lowest proportion of the essential post-implantation self-care steps that a patient should be able to perform unaided to be regarded as safe for discharge, and this threshold was fixed in the study protocol before recruitment began. We acknowledge that a threshold derived by expert consensus is a pragmatic clinical judgement rather than an empirically validated cut-point, and that the categorical competence results should therefore be read alongside the continuous scores, which do not depend on any threshold.

#### Tool IV – Self-Care Behaviours Scale (modified from Rayamajhi et al., 2021 [13])

10 domains, each scored always = 2, sometimes = 1, or never = 0 (total 0–20); higher scores denote more consistent self-care. Positive self-care behaviour was defined as ≥ 70% (≥ 14 points). This cut-off was likewise established a priori, before data collection, by the same expert-consensus process used for the Tool III threshold, and was set deliberately at the same proportion so that the two behavioural instruments would be interpreted on a common metric. It was not derived from previous literature or from an institutional standard, and it does not represent an empirically validated minimal clinically important difference; the developers of the original scale did not specify a cut-point, and none has been established subsequently. As with Tool III, we therefore present the categorical classification as a descriptive aid and base the inferential analyses on the continuous scores. Because Tool IV shares its domain structure with Tool III, the two instruments capture related but methodologically distinct constructs directly observed competence versus self-reported behaviour a point returned to in the interpretation of their correlation and in the Limitations.

The English-language source instruments were translated into Arabic and back-translated using a standard forward–backward procedure: two bilingual nursing academics independently produced forward translations, which were reconciled by consensus and back-translated into English by an independent bilingual translator blinded to the original instruments. Discrepancies were resolved by consensus and the reconciled Arabic version was pre-tested for comprehensibility on 10 patients who were not part of the study sample, are separate from the seven pilot participants described under Participants, and whose data were not included in the analysis. Content validity of the translated tools was established by a panel of five experts in critical care and medical-surgical nursing (content validity index > 0.85). Internal consistency was evaluated in the present study sample (*N* = 70): the knowledge questionnaire (Tool II) yielded Cronbach’s α = 0.930 across 20 items; the practice-competence observational checklist (Tool III) yielded Cronbach’s α = 0.936 across 50 items; and the self-care behaviours scale (Tool IV) yielded Cronbach’s α = 0.745 across 10 domains. These coefficients indicate acceptable to excellent internal consistency; they do not, however, establish test–retest stability or discriminant validity, neither of which was assessed.

### Data-collection procedure

#### Phase 1 – Assessment (baseline)

All participants completed Tools I–IV.

#### Phase 2 – Planning

Informed by baseline deficits, the theoretical framework, and the literature, the empowerment protocol and booklet were developed and validated.

#### Phase 3 – Implementation (study group only)

Four individual sessions were delivered as described above, followed by weekly telephone reinforcement during the first month after implantation.

#### Phase 4 – Evaluation

Post-intervention assessment was performed immediately after the final session, and follow-up assessment at one month post-discharge (via outpatient-clinic visit, or home visit for non-attenders).

- **Session 1 (Pre-procedure)**: Focused on baseline knowledge, basic cardiac anatomy, pacemaker function, and fear reduction.
- **Session 2 (Post-procedure Day 1)**: Addressed wound care, lead stabilization, infection prevention, and upper extremity range-of-motion precautions.
- **Session 3 (Post-procedure Day 2)**: Trained patients on daily manual pulse measurement, identification of cardiac symptoms, and electromagnetic interference safety.
- **Session 4 (Discharge Day)**: Established individualized self-care action plans and emergency response protocols.

Following discharge, intervention fidelity and adherence to the self-care plan were reinforced by weekly structured telephone contacts during the first month post-implantation (four calls in total). These calls formed part of the intervention and were delivered only to the study group; control participants received no equivalent contact. Delivery of every session and call was documented contemporaneously on a fidelity checklist. All 140 planned sessions were delivered (140/140, 100%) and 136 of the 140 planned telephone contacts were completed (97.1%); the four missed calls were distributed across four different participants, each of whom received the remaining three calls, and no participant missed more than one call. All 35 study-group participants therefore received the complete face-to-face component of the protocol and at least three of the four planned telephone contacts.

### Statistical analysis

Data were analysed using SPSS version 26 (IBM Corp., Armonk, NY, USA). Distributional assumptions were examined with the Shapiro–Wilk test and visual inspection of Q–Q plots; continuous variables were consistent with normality (*p* > 0.05) and are expressed as mean ± standard deviation, while categorical variables are presented as frequencies and percentages (n, %). Homogeneity of variance was assessed with Levene’s test.

Outcomes and multiplicity. Knowledge, practice competence and self-care behaviour were designated a priori as three co-primary outcomes, reflecting the three domains the empowerment protocol was built to address; no single outcome was ranked above the others and no secondary outcomes were defined. Because three co-primary outcomes were tested, the family-wise type I error rate was controlled by applying a Bonferroni-adjusted significance threshold of α = 0.017 (0.05/3) to the three principal group × time interaction tests, which constitute the primary statistical comparison. The same adjusted threshold was applied to the post-intervention and follow-up between-group contrasts for each outcome. The following analyses were pre-specified: the three group × time interactions; the between-group contrasts at post-intervention and one-month follow-up. The following analyses were exploratory and are reported without adjustment and without inferential claim: the knowledge–self-care correlations, the within-group paired contrasts across time points, and the categorical distributions of knowledge level and competence level. Exploratory results are interpreted descriptively only.

Repeated-measures analyses. A 2 (group) × 3 (time) mixed-model repeated-measures ANOVA was fitted for each outcome, with group as the between-subjects factor and time as the within-subjects factor. Sphericity was tested with Mauchly’s test; where it was violated the Greenhouse–Geisser correction was applied. All F statistics, degrees of freedom and p values reported in Table 5 for knowledge and practice are the Greenhouse–Geisser-corrected values, with fractional degrees of freedom and the corresponding epsilon reported alongside; for self-care behaviour, sphericity was not violated and the uncorrected values are reported. Partial eta squared (η²p) is reported as the effect-size index. Significant interactions were decomposed in two ways: by testing the simple effect of group at each time point using independent t-tests, and by testing the simple effect of time within each group using Bonferroni-corrected pairwise contrasts across the three time points. Independent and paired t-tests were used for between- and within-group comparisons respectively, and for the between-group contrasts mean differences with 95% confidence intervals and Cohen’s d were computed.

Normality of residuals and homogeneity of error variance were checked by inspection of residual plots and Levene’s test respectively. Pearson’s correlation coefficient was used for the exploratory association between total knowledge and self-care scores. Statistical significance was set at *p* < 0.05 for exploratory analyses and at the Bonferroni-adjusted α = 0.017 for the co-primary comparisons. Data completeness was examined before analysis; a complete-case strategy was used, and no missing values or drop-outs occurred across the three evaluation phases (*N* = 70; 100% retention), supported by structured mobile reminders and scheduled clinic follow-up.

### Ethical considerations

Approval was obtained from the Scientific Research Ethics Committee, Faculty of Nursing, Tanta University (approval code 817-10-2025), and the study was conducted in accordance with the Declaration of Helsinki. Written informed consent was obtained from all participants after explanation of the study purpose, procedures, risks (none anticipated), and benefits. Participants were assured of confidentiality and of their right to withdraw at any time without any effect on their care. As a non-randomised quasi-experimental evaluation of an educational intervention, formal prospective clinical-trial registration was not undertaken at inception; the study was subsequently registered retrospectively on ClinicalTrials.gov (NCT07743346) in accordance with journal policy, and was conducted and reported prospectively throughout.

## Results

### Participant characteristics

The final sample comprised 70 participants (35 per group), with no attrition. Mean age was 58.81 ± 7.47 years in the control group and 57.34 ± 7.99 years in the study group (*p* = 0.383). Males predominated (65.7% of controls, 57.1% of the study group; *p* = 0.461). The groups did not differ significantly in education (*p* = 0.972), marital status (*p* = 0.640), or residence (*p* = 0.632) (Table 1).

**Table 1:** Sociodemographic characteristics of the study and control groups (*n* = 70)

Variable	Control (*n* = 35), n (%)	Study (*n* = 35), n (%)	χ² / t	*P*
**Age group (years)**			2.167	0.539
40–50	4 (11.4)	6 (17.1)		
51–60	12 (34.3)	16 (45.7)		
61–70	17 (48.6)	12 (34.3)		
71–80	2 (5.7)	1 (2.9)		
Mean ± SD	58.81 ± 7.47	57.34 ± 7.99	t = 0.878	0.383
**Sex**			0.543	0.461
Male	23 (65.7)	20 (57.1)		
Female	12 (34.3)	15 (42.9)		
**Education**			0.236	0.972
Illiterate	8 (22.9)	9 (25.7)		
Read & write	10 (28.6)	11 (31.4)		
Intermediate	10 (28.6)	9 (25.7)		
High	7 (20.0)	6 (17.1)		
**Marital status**			1.687	0.640
Single	2 (5.7)	1 (2.9)		
Married	22 (62.9)	24 (68.6)		
Widowed	9 (25.7)	6 (17.1)		
Divorced	2 (5.7)	4 (11.4)		
**Residence**			0.229	0.632
Rural	17 (48.6)	19 (54.3)		
Urban	18 (51.4)	16 (45.7)		

Categorical variables compared with the chi-square test; age compared with the independent t-test

Baseline clinical characteristics are reported in Table 2. Dual-chamber (DDD) pacing was the more common configuration, accounting for 71.4% of the control group and 68.6% of the study group, while single-chamber (VVI/AAI) devices represented 28.6% and 31.4% respectively; the groups did not differ significantly (χ² = 0.068, *p* = 0.794). Hypertension was the most prevalent comorbidity across the whole sample, affecting 57.1% of controls and 62.8% of the study group (χ² = 0.238, *p* = 0.626), followed by ischaemic heart disease in 40.0% and 34.3% respectively (χ² = 0.245, *p* = 0.621), diabetes mellitus in 40.0% and 37.1% (χ² = 0.060, *p* = 0.806), arrhythmia in 25.7% and 37.1% (χ² = 1.061, *p* = 0.303) and chronic kidney disease in 11.4% and 8.6% (χ² = 0.159, *p* = 0.690). The groups did not differ significantly on any recorded clinical characteristic.

**Table 2:** Baseline clinical characteristics of the study and control groups (*n* = 70)

Variable	Control (*n* = 35), n (%)	Study (*n* = 35), n (%)	χ²	*P*
**Pacing mode**				
Single chamber (VVI/AAI)	10 (28.6)	11 (31.4)	0.068(0.794)	
Dual chamber (DDD)	25 (71.4)	24 (68.6)		
**Comorbidities**				
Hypertension	20 (57.1)	22 (62.8)	0.238(0.626)	
Diabetes mellitus	14(40)	13(37.1)	0.060(0.806)	
Arrhythmia	9 (25.7)	13 (37.1)	1.061(0.303)	
Ischaemic heart disease	14 (40)	12(34.3)	0.245(0.621)	
Chronic kidney disease	4 (11.4)	3 (8.6)	0.159(0.690)	

Categorical variables compared with the chi-square test; for pacing mode and each comorbidity the chi-square statistic is shown with its p value in parentheses

### Clinical diagnoses

**Fig. 2:**
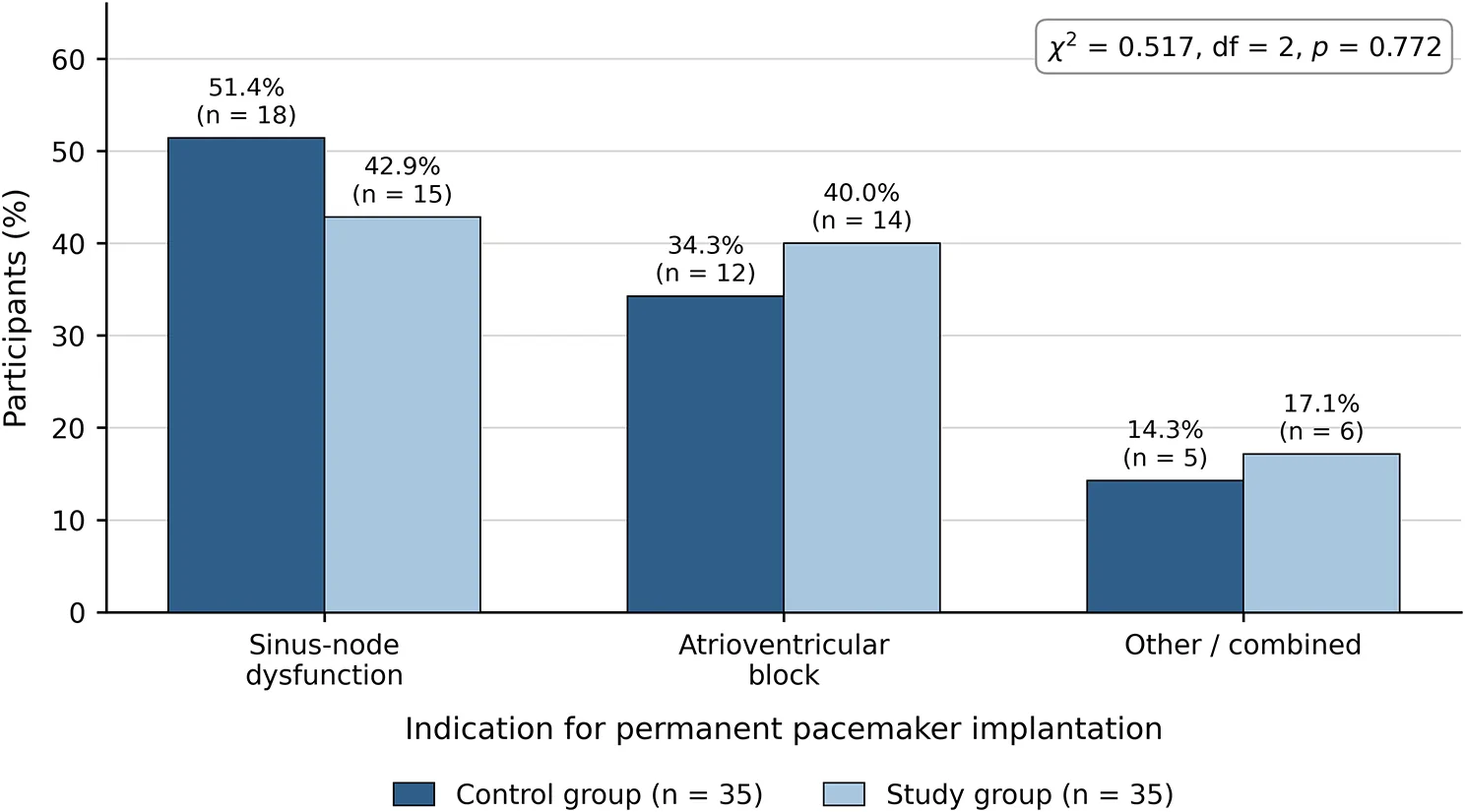
Indication for permanent pacemaker implantation in the study and control groups (Figure file submitted separately)

Pacemaker implantation was indicated by a documented rhythm or conduction abnormality in every participant. The single most common indication was sinus-node dysfunction (sick sinus syndrome, including tachycardia–bradycardia syndrome) in 51.4% of controls and 42.9% of the study group. Atrioventricular block was the indication in 34.3% of controls and 40.0% of the study group, and other or combined conduction abnormalities accounted for the remainder (14.3% and 17.1% respectively). The full distribution is shown in Fig. 2 and did not differ significantly between the groups (χ² = 0.517, df = 2, *p* = 0.772). Patients whose underlying cardiac diagnosis was ischaemic heart disease or heart failure did not receive a device for those diagnoses per se; in every such case the device was implanted for a coexisting guideline-based rhythm or conduction abnormality, which is the indication classified in Fig. 2. Supplementary file.

### Knowledge outcomes

At baseline, knowledge was poor in both groups (68.6% of controls and 65.7% of the study group). Post-intervention, the study group improved markedly 80.0% achieved good knowledge—and this was largely sustained at follow-up (77.1%). The control group improved only modestly (37.1% good post-intervention; 28.6% at follow-up) (Table 3).

**Table 3:** Distribution of knowledge level across study phases

Group	Phase	Poor, *n* (%)	Fair, *n* (%)	Good, *n* (%)
Control	Pre	24 (68.6)	3 (8.6)	8 (22.9)
	Post	20 (57.1)	2 (5.7)	13 (37.1)
	Follow-up	21 (60.0)	4 (11.4)	10 (28.6)
Study	Pre	23 (65.7)	5 (14.3)	7 (20.0)
	Post	5 (14.3)	2 (5.7)	28 (80.0)
	Follow-up	7 (20.0)	1 (2.9)	27 (77.1)

### Practice competence (observed)

At baseline, most participants were non-competent (94.3% of controls, 91.4% of the study group). Post-intervention, 85.7% of the study group became competent, compared with only 17.1% of controls (*p* < 0.001). Gains in the study group declined slightly by follow-up but remained well above those of controls (Table 4).

**Table 4:** Practice-competence levels across study phases

Group	Phase	Competent, *n* (%)	Non-competent, *n* (%)	Test statisticᵃ	*P*
Control	Pre	2 (5.7)	33 (94.3)	Q = 4.800	0.091
	Post	6 (17.1)	29 (82.9)		
	Follow-up	4 (11.4)	31 (88.6)		
Study	Pre	3 (8.6)	32 (91.4)	Q = 52.074	< 0.001
	Post	30 (85.7)	5 (14.3)		
	Follow-up	29 (82.9)	6 (17.1)		

ᵃ Cochran’s Q test for within-group change over time

### Mean scores and repeated-measures ANOVA

Table 5 presents mean scores and the repeated-measures ANOVA results. The study group scored higher than the control group on all three co-primary outcomes at post-intervention and follow-up, with large effect sizes (η²p 0.423–0.685). The group × time interaction was significant for every outcome at the Bonferroni-adjusted family-wise threshold (α = 0.017), indicating that the trajectories of change differed between the two groups. Because allocation was not randomised, these interactions describe differing trajectories between two sequentially recruited cohorts and should not be read as isolating the causal effect of the protocol.

**Table 5:** Mean scores and repeated-measures ANOVA for knowledge, practice, and self-care behaviour

Outcome	Group	Pre	Post	Follow-up	Group × time F (corrected df)	Greenhouse–Geisser ε	*P*	η²*p*
Knowledge (0–20)	Control	5.57 ± 2.47	7.46 ± 5.60	7.29 ± 4.62	F(1.43, 97.38) = 148.09	0.716	< 0.001	0.685
	Study	6.51 ± 2.45	15.31 ± 3.46	15.03 ± 3.88				
Practice (0–50)	Control	14.91 ± 3.33	18.54 ± 12.21	16.46 ± 10.87	F(1.59, 108.12) = 120.34	0.795	< 0.001	0.639
	Study	16.29 ± 2.87	41.17 ± 10.00	40.00 ± 8.90				
Self-care behaviour (0–20)	Control	12.86 ± 2.30	15.00 ± 3.10	13.31 ± 3.10	F(2, 136) = 49.85	0.557	< 0.001	0.423
	Study	13.63 ± 3.11	19.00 ± 3.23	17.43 ± 3.34				

For knowledge and practice, the F statistics, degrees of freedom and p values shown are the Greenhouse–Geisser-corrected values, reported as fractional degrees of freedom with the corresponding epsilon (ε); for self-care behaviour, sphericity was not violated and the uncorrected values are shown (ε = 0.557)

Decomposition of the significant interactions and the post-hoc procedure are described in the Statistical analysis section. All three interactions remain significant at the family-wise threshold α = 0.017.

### Between-group effect sizes

To quantify the magnitude of the intervention effect, between-group mean differences (study minus control) with 95% CIs and Cohen’s d were computed at the post-intervention and follow-up assessments (Table 6). All contrasts represent large effects, with confidence intervals excluding the null and all p values below the family-wise threshold of 0.017.

**Table 6:** Between-group differences, 95% confidence intervals, and effect sizes

Outcome	Time point	Mean difference (study − control)	95% CI	Cohen’s d
Knowledge	Post	7.85	5.67 to 10.03	1.69
Knowledge	Follow-up	7.74	5.74 to 9.74	1.81
Practice	Post	22.63	17.40 to 27.86	2.03
Practice	Follow-up	23.54	18.89 to 28.19	2.37
Self-care	Post	4.00	2.52 to 5.48	1.26
Self-care	Follow-up	4.12	2.61 to 5.63	1.28

### Correlation between knowledge and self-care

In the study group, a strong positive correlation between knowledge and self-care emerged post-intervention (*r* = 0.946, *p* < 0.001) and remained significant, though attenuated, at follow-up (*r* = 0.595, *p* < 0.001). No significant correlations were found in the control group at any phase (Table 7). These correlations were exploratory and are not adjusted for multiplicity. Because the knowledge and self-care instruments share overlapping content domains and were administered close in time, the very high immediate coefficient most plausibly reflects shared method and construct variance rather than a purely substantive association, and it should be interpreted with caution (see Discussion and Limitations).

**Table 7:** Pearson correlation between total knowledge and self-care scores

Phase	Control *r*	Control *p*	Study *r*	Study *p*
Pre-intervention	0.180	0.300	0.202	0.246
Post-intervention	0.235	0.174	0.946	< 0.001
Follow-up	0.268	0.120	0.595	< 0.001

## Discussion

This quasi-experimental study evaluated a structured, theory-informed, nurse-led empowerment protocol targeting knowledge, practical skills, and self-care behaviours in patients with permanent pacemakers. Participation in the protocol was associated with statistically significant improvements across all three co-primary outcomes relative to routine hospital care alone, with large effect sizes (η²p 0.423–0.685; between-group Cohen’s d 1.26–2.37) and significant group × time interactions that survived adjustment for multiplicity. Interpreted through the study’s framework, these results are consistent with the proposition that structured mastery experiences and reinforcement raise self-efficacy, which in turn is expressed as improved self-care [9, 10]. Because the two groups were recruited in consecutive calendar periods rather than randomised concurrently, the design supports an inference of association under a quasi-experimental framework and not a causal inference equivalent to that of a randomised controlled trial.

### Improvement in knowledge

Post-intervention, the study group’s mean knowledge score reached 15.31 of 20, compared with 7.46 in the control group a between-group difference of 7.85 points (95% CI 5.67–10.03; d = 1.69). This pattern is consistent with Hassan et al. [14], who reported comparable gains following a nursing care protocol for pacemaker patients. The largely preserved knowledge at one-month follow-up (15.03) suggests that the illustrated booklet and interactive sessions supported retention, in line with Ibrahim et al. [5], and is congruent with Bandura’s account of verbal persuasion and reinforcement consolidating learning [9].

### Enhancement of practical self-care skills

Baseline practice competence was strikingly low (< 12% competent in both groups), reflecting inadequate preparation under routine care. After the protocol, 85.7% of the study group became competent, compared with only 17.1% of controls (between-group difference 22.63 points; 95% CI 17.40–27.86; *d* = 2.03). This echoes Khalil et al. [15], who reported that satisfactory practice rose from 2.9% to 65.7% following an educational intervention. The larger post-intervention competence observed here may reflect the emphasis on demonstration and return demonstration—mastery experiences that are the strongest source of self-efficacy for psychomotor skills such as pulse measurement and wound inspection [9, 16].

### Self-care behaviour and the knowledge–behaviour link

Self-care behaviour improved significantly more in the study group (between-group difference 4.00 points; 95% CI 2.52–5.48; *d* = 1.26), supporting the framework’s prediction that raising knowledge and self-efficacy translates into behaviour, consistent with Vatmasari et al. [17]. The very high post-intervention correlation between knowledge and self-care (*r* = 0.946) should not, however, be read as strong substantive evidence of a knowledge–behaviour mechanism: the two instruments share content domains and were administered close in time, so the coefficient is likely inflated by common-method and construct overlap. The attenuation of the correlation at one month (*r* = 0.595), together with the slight decline in both knowledge and self-care between post-intervention and follow-up, points to the value of planned booster sessions.

### Comparison with routine care

The minimal improvement in the control group indicates that routine discharge instructions typically verbal, unstructured, and lacking reinforcement are unlikely to be sufficient for this population. This observation is clinically important, as many hospitals in low- and middle-income countries continue to rely on such practices. Notably, a structured protocol delivered by a single nurse was associated with large gains, suggesting the approach may be feasible even in resource-constrained settings [13]. That said, the study group received four face-to-face sessions and four telephone contacts that the control group did not, so part of the between-group difference may reflect the nonspecific effect of additional professional attention rather than the educational content alone; this is considered further below.

### Clinical relevance of the observed changes

Large standardised effects do not by themselves establish clinical importance, and no minimal clinically important difference (MCID) has been published for any of the three instruments used here; none of them is anchored to a patient-reported or clinical outcome. The clinical relevance of the observed changes must therefore be judged against the content of the items rather than against an established threshold. On that basis, several of the changes appear substantively meaningful. The mean gain of 8.8 points on the 20-item knowledge questionnaire moved the study group from a mean below the ‘poor’ boundary to a mean within the ‘good’ band, meaning that the average participant moved from answering roughly a third of items correctly to answering roughly three-quarters correctly, including items on wound infection warning signs and electromagnetic-interference precautions. The proportion of the study group able to perform at least 70% of the observed self-care steps unaided rose from 8.6% to 85.7%: expressed as an absolute risk difference against the control group at post-intervention this corresponds to 68.6% points, or a number needed to treat of approximately 1.5 patients to produce one additional competent patient. Skills such as accurate manual pulse measurement and recognition of incision infection are prerequisites for the early detection of pacemaker complications, so a change of this magnitude is plausibly consequential. Against this, the 4.0-point mean gain on the 20-point self-care behaviours scale corresponds to a shift of roughly two domains from ‘sometimes’ to ‘always’, which is more modest, was partly eroded by one month, and was self-reported. Most importantly, no clinical endpoint infection, lead dislodgement, unplanned readmission, device-related complication or mortality was measured, so the study cannot demonstrate that these behavioural gains translate into better clinical outcomes. Establishing MCIDs for these instruments, and linking them to hard endpoints, is a priority for future work.

### Limitations

Several limitations should be acknowledged, the first of which is central to the interpretation of every result reported here. First, allocation was not random and was completely determined by calendar period: the entire control group was recruited and assessed between December 2025 and February 2026 and the entire intervention group between February and April 2026. Group membership is therefore perfectly confounded with time, and any change occurring between the two periods in nursing or medical staffing, discharge education, implantation practice, device selection, patient case-mix, referral patterns, hospital policy or the availability of educational material could have contributed to the observed differences. We did not conduct a formal contemporaneous audit of such changes, and the brevity of the study period is not in itself evidence that the clinical environment was stable; it merely constrains how much could have changed. This confounding cannot be removed by statistical adjustment. Although the two groups did not differ significantly on any recorded baseline sociodemographic or clinical characteristic, comparability on measured variables is not evidence of comparability on unmeasured ones, and no covariate adjustment can correct for unmeasured secular change; the findings should accordingly be read as an association observed under a quasi-experimental design rather than as causal evidence. Second, the study group received four structured face-to-face sessions plus four telephone contacts, while the control group received no attention-matched contact whatever; the observed effects may therefore partly reflect nonspecific professional attention (a Hawthorne/attention effect) rather than the specific educational content, and future trials should include an attention-matched comparator. Third, outcome assessment was not blinded to group allocation. Because practice competence was measured by direct observation and the intervention itself consisted partly of return demonstration of the very skills scored, the potential for observer bias in the practice outcome is substantial; the explicit, pre-specified scoring criteria reduce but do not eliminate it, and no independent second assessor scored a sample of observations, so inter-rater reliability is unknown. Fourth, the observed-practice (Tool III) and self-reported self-care (Tool IV) instruments share their domain structure, which limits their discriminant validity and inflates the observed knowledge–behaviour correlation; the two constructs should be measured with more clearly separated instruments in future work. Fifth, no minimal clinically important difference exists for any of the three instruments, and no clinical endpoint was measured, so the clinical importance of the changes reported here is inferred from item content rather than demonstrated. Sixth, social-desirability bias may have affected the self-reported self-care scores, and this bias would be expected to act in the direction of the hypothesis in the unblinded intervention group. Seventh, the effect size used for the sample-size calculation was derived from a pilot of only seven participants and is correspondingly imprecise; internal-consistency coefficients were estimated in the study sample itself, and test–retest stability was not assessed. Eighth, three co-primary outcomes were analysed and a Bonferroni-adjusted family-wise threshold was applied to the principal comparisons, but the correlational and within-group analyses were exploratory and unadjusted, so residual inflation of type I error cannot be excluded for those results. Ninth, the single-centre, tertiary-hospital setting limits generalisability, and the trial was registered retrospectively. Finally, follow-up extended only to one month, so longer-term sustainability is unknown; the apparent consistency of gains across the literacy range represented should be confirmed in a pre-specified stratified analysis rather than inferred from the overall effect size.

### Implications for practice, policy, and research

#### Clinical practice

Hospitals should adopt systematic, theory-informed empowerment protocols incorporating individualised interactive education, demonstration and return demonstration, and illustrated take-home materials, and nurses should be trained in patient-empowerment and self-efficacy-building techniques.

#### Policy

Routine, unstructured discharge instructions should be replaced by structured protocols, with documentation of patient comprehension and skill demonstration before discharge.

#### Research

Future research should include: randomised controlled trials with blinded outcome assessment, an attention-matched comparator, and longer follow-up (e.g., six months); multicentre studies to assess generalisability across healthcare systems; discriminant-validity work to separate observed competence from self-reported behaviour; cost-effectiveness analyses of empowerment protocols; evaluation of digital (mobile-health) reinforcement strategies; and exploration of family-caregiver involvement in sustaining self-care behaviours.

## Conclusions

In this non-randomised, single-centre study, participants who received a structured, theory-informed, nurse-led empowerment protocol showed substantial and partially sustained gains in knowledge, practical skills, and self-care behaviours, whereas the corresponding gains observed under routine hospital care alone were minimal. Because the two groups were recruited in consecutive calendar periods rather than randomised concurrently, and because outcome assessment was unblinded, these results are best interpreted as an association observed under a quasi-experimental design rather than as causal evidence equivalent to that of a randomised controlled trial. They nonetheless provide a reasonable basis for evaluating systematic, interactive empowerment programmes—incorporating individualised education and scheduled reinforcement—as an addition to routine post-implantation nursing care, and for testing them in randomised, attention-controlled, multicentre trials with blinded assessment and longer follow-up.

## Data Availability

The datasets generated and analysed during the current study are not publicly available due to the confidentiality of participants’ personal and clinical information, but are available from the corresponding author on reasonable request.

## Abbreviations

ANOVA: Analysis of variance
CI: Confidence interval
CVI: Content validity index
GG: Greenhouse–Geisser
MCID: Minimal clinically important difference
NNT: Number needed to treat
NYHA: New York Heart Association
PM: Permanent pacemaker
RM-ANOVA: Repeated-measures analysis of variance
SD: Standard deviation
SPSS: Statistical Package for the Social Sciences
TREND: Transparent Reporting of Evaluations with Nonrandomized Designs
η²p: Partial eta squared
